# The Results of Operation in Fifty-Four Cases of Cancer of the Breast

**Published:** 1902-12

**Authors:** Charles A. Morton

**Affiliations:** Professor of Surgery at University College, Bristol; Surgeon to the Bristol General Hospital and to the Royal Hospital for Sick Children and Women


### THE RESULTS OF OPERATION IN FIFTY-FOUR CASES OF CANCER OF THE BREAST.

BY

CHARLES A. MORTON, F.R.C.S.,

*Professor of Surgery at University College, Bristol; Surgeon to the Bristol General Hospital and to the Royal Hospital for Sick Children and Women.*

---

THE only way to form a true estimate of the value of operation for cancer of the breast is to study the complete records of a large number of consecutive cases. That this may be done, I have prepared a table of my cases, and I am glad to say I have been better able to discover the proportion of recurrences than one might expect, for there is only one case which I have not been able to trace up to the present time or until recurrence was noted.

This series of 54 cases includes all the cases of breast cancer I have operated on, but does not include a few cases I have operated on for sarcoma, or any cases of so-called "duct-cancer," which is a much less malignant type of growth than ordinary breast scirrhus, for I have not operated on any case of the kind. I have had no death from the operation, or for some months after, nor have I had any death in the few cases of operation for sarcoma of the breast. Hence we may assure patients that the risk is very slight, though it is so extensive an operation.

There are 30 cases operated on up to November, 1899, and therefore cases in which the patient might be free from recurrence for three years or longer. Such cases are generally regarded by surgeons as cures. Out of these 30 cases, nine have had no recurrence for three years or longer, *i.e.*, nearly one in every three cases is cured. In two of these nine cases it is eight years since the operation, and in two five years. In all the nine cases the appearance of the growth was typical, and in all but two the opinion as to the nature of the growth formed from its appearance was confirmed either by microscopic section (5 cases) or the presence of definite growth in the glands (5 cases); only in two cases (19) was the evidence as to the nature of the growth from its naked-eye appearance not corroborated by either microscopic section, or the presence of growth in the glands.

Amongst these 30 cases were several in which the growth was very extensive, and in only three could it be said to be small, *i.e.* the size of a walnut or smaller. Of the 21 cases in which recurrence took place, one had no recurrence for three years, and then had two small recurrences removed, but was free from recurrence when she died suddenly of heart disease five years after operation: another had no sign of recurrence for three years, and then recurrence was discovered and removed this year: she has now another recurrence, but is in good general health. In another case (28) there is now, I think, a small subcutaneous recurrence three years after the operation. As I have not seen her in the last two years, I cannot say how long it has been there; but there is every prospect of its removal, with another long interval of non-recurrence or of actual cure.

Out of the 14 cases operated on between November, 1899, and November, 1901, half are now free from recurrence a year or more after the operation. One case operated on 2 years and 5 months, one 2 years and 3 months, one 1 year and 11 months, one 1 year and 10 months, one 1 year and 8 months, one 1 year and 5 months, and two 1 year ago, are now all free from recurrence. One died from secondary growth two years after operation without local recurrence. These statistics look as if a decidedly larger proportion of cases of cure would eventually be found in this series, for Mr. Watson Cheyne[1] has shown that of 20 cases in which no recurrence had been found at the end of a year, 17 were free from recurrence three years after. Of the cases operated on during the last year, it is too early yet to enquire as to results. Any recurrence I know of I have stated.

I think then we may promise patients not only a remarkable freedom from risk, considering the extent of the operation, but a fair chance of non-recurrence for many years—and if there is no recurrence for many years, probably of non-recurrence altogether. I cannot myself regard cases of non-recurrence for

[1] *Lancet*, 1899, i. 757.

three years as certain cures. I admit that they are quite unlikely to get any recurrence after that time, but we must recognise the fact that now and then they do. I have already referred to two such cases of my own. In one the second operation disclosed more extensive disease buried under the pectorals, just below the inner half of the clavicle, than I expected to find, and possibly when I examined her at the end of the third year after the first operation I may have overlooked this. In the other case there is no doubt small subcutaneous nodules began to recur only after the third year, but they were removed, and the patient lived for two years after the first recurrence and had none at the time of her death. Several other cases of recurrence after three years have been recorded by other surgeons. That cases in which there is no recurrence for three years are very unlikely to get any recurrence later is shown by Mr. Watson Cheyne's[1] 12 cases, in which recurrence had not taken place three years after operation, and three years later nine were still free from recurrence, one had died of a cancerous growth in the intestine (probably primary), one of bronchitis, and one could not be traced.

In only one of my cases in which there has been no recurrence for three years or more was the growth smaller than a hen's egg, but the skin or pectoral muscle was not involved in any, nor were the glands extensively affected. In every case but one an incision was made into the growth before removal to verify the diagnosis, but the knife with which this incision was made was not used again. Unless the growth is large, I have now abandoned this practice for fear of infecting divided lymphatics with cancer cells, and I dissect out the tumour before cutting into it or remove one of the axillary glands for inspection if they are enlarged; but the fact that eight of the cases did so well after this preliminary incision, without previous removal of the growth, shows that it is not such a dangerous practice as some surgeons now maintain.

My records show that very large growths, often involving the muscles and very extensively the glands, are, as might be expected, frequently followed by quick recurrence. One case of

[1] *Loc. cit.*

| NO. | NAME AND AGE. | DATE OF OPERATION. | SENT TO ME BY. (Where the patient was not specially sent to me, the name of Hospital in which operation done is given.) | CONDITION OF GROWTH. | RESULT. |
|---|---|---|---|---|---|
| | | | | *Cases operated on before Nov.,* | 1899. |
| 1 | Mrs. R. 60 | April, 1894 | General Hospital.. | Very large, involving pectoral and skin. | Recurred in six months: position unknown. |
| 2 | Mrs. A. 53 | Aug., 1894 | Dr. Burroughs ... | Very large and large mass of glands | Rapid recurrence: position unknown. |
| 3 | S. J. 55 | Jan., 1894 | General Hospital.. | Size of orange. Two glands ... | Writes to say now well, eight years after operation. Micro. exam. showed carcinoma. |
| 4 | E. F. 46 | Jan., 1894 | General Hospital.. | Size hen's egg. Scirrhus growth in one gland. | Writes to say now well, eight years after operation. |
| 5 | M. R. 51 | July, 1893 | Dr. A. Carr ... ... | Moderate size. Scirrhus glands. | No recurrence for three years, then two operations for small nodules under skin. Sudden death from heart disease five years after operation without further recurrence. |
| 6 | M. V. 68 | Nov., 1894 | General Hospital.. | Size hen's egg ... ... ... | Recurrent nodules in subcutaneous tissue, not involving pectoral muscle, nine months after operation. |
| 7 | E. C. 59 | Oct., 1895 | Mr. A. W. Peake.. | Size hen's egg. Growth in glands. Micro. section showed cancer. | Writes to say now well, seven years after operation. |
| 8 | J. M. 36 | Jan., 1897 | Dr. T. Freeman ... | Two small scirrhus growths ... | Recurrent nodules in seven months in skin, too numerous to remove. |
| 9 | E. P. 54 | Mar., 1897 | General Hospital.. | Moderate size and skin involved . | Recurrence seen in fifteen months in supra-clavicular glands and sternum. |
| 10 | G. G. 53 | April, 1897 | General Hospital.. | Moderate size ... ... ... | Writes to say now well, five and half years after operation. |
| 11 | M. M. | May, 1897 | General Hospital.. | Extensive growth involving pectoral, and large mass of glands. | Died at home a few months after operation. ? recurrence. |
| 12 | E. C. 53 | May, 1897 | Mr. Churton Pauli | Moderate size, and with skin involved. | Recurrence observed after a year: position unrecorded. |
| 13 | S. P. 54 | Oct., 1897 | Dr. J. Young ... | Size hen's egg. Micro. section showed cancer. | Dr. Young reports now well, five years after operation. |
| 14 | Miss S. 30 | Oct., 1897 | Mr. Windsor Aubrey | Large multiple growths, and extensive gland growth. | Recurrence observed sixteen months later: position unknown. |

| NO. | NAME AND AGE. | DATE OF OPERATION. | SENT TO ME BY. (Where the patient was not specially sent to me, the name of Hospital in which operation done is given.) | CONDITION OF GROWTH. | RESULT. |
|---|---|---|---|---|---|
| 15 | M. M. 75 | Sept., 1897 | Dr. L. Lees ... ... | Small growth of doubtful nature when removed from breast, but found on section to be cancer. | Still alive. Writes to say she has had recurrence last two years. |
| 16 | A. A. 45 | Feb., 1898 | General Hospital.. | Size walnut ... ... ... ... | Recurrence found one year later in subcutaneous tissue and surface of pectoral muscle, and removed. Then recurred in chest wall, and finally in other breast. |
| 17 | Mrs. J. 54 | Feb., 1898 | Mr. A. W. Peake.. | Moderate size. Skin involved and ulcerating. | Small nodule in "chest wall" removed fifteen months after first operation. Died from double pleural effusion two years and three months after operation. |
| 18 | S. B. 42 | Feb., 1898 | General Hospital.. | Size orange ... ... ... ... | Cannot be traced. |
| 19 | E. L. 44 | Dec., 1898 | General Hospital.. | Size hen's egg ... ... ... | Writes to say now well, three years and eleven months since operation. |
| 20 | Mrs. W. | Jan., 1899 | Dr. Ambrose ... | Moderate size ... ... ... | Died from effects of alcohol without recurrence some months after operation. |
| 21 | M. C. 56 | Jan., 1899 | General Hospital.. | Size orange and many glands .. | Recurred in six months as nodule in skin. |
| 22 | R. E. 52 | Jan., 1899 | General Hospital.. | Moderate size, involving skin, and another small growth in breast. | No recurrence for three years when examined by me; then recurrence found in supra-clavicular glands, and deeply beneath both pectorals just under inner end of clavicle. |
| 23 | J. S. 49 | April, 1899 | Mr. Brew ... .. | Size orange and scirrhus glands. Micro. section showed scirrhus. | Mr. Brew reports no recurrence now, three years and six months after operation. |
| 24 | H. A. 54 | May, 1899 | Mr. Brew ... ... | Size of orange ... ... ... | Recurred in about six months. |
| 25 | E. M. 59 | May, 1899 | General Hospital. | Very extensive, involving pectoral, skin, and large mass of glands. | Recurred in a few months in skin and supra-clavicular glands |
| 26 | Mrs. P. 60 | July, 1899 | Dr. Belfield ... ... | Small growth ... ... ... | Dr. Belfield reports no local recurrence, but died of growths. in spine and stomach two years and eleven months after operation. |
| 27 | E. S. 43 | Aug., 1899 | General Hospital.. | Size of hen's egg ... ... ... | Rapid recurrence. |
| 28 | C. D. 66 | Sept., 1899 | Mr. F. Peake ... | Moderate size, and skin involved and ulcerating. | Recurrence only just discovered, and of small size, three years and two months after operation. |

| NO. | NAME AND AGE. | DATE OF OPERATION. | SENT TO ME BY. (Where the patient was not specially sent to me, the name of Hospital in which operation done is given.) | CONDITION OF GROWTH. | RESULT. |
|---|---|---|---|---|---|
| 29 | L. H. 73 | June, 1899 | Mr. F. Peake ... | Very large growth, with degeneration cyst. Micro. section showed branching columns of epithelial cells. | Examined by me Nov. 5th; no recurrence, three years and three months after operation. |
| 30 | Mrs. F. 37 | Nov., 1899 | Dr. Wilding ... | Small size, and small but scirrhus glands. | Writes to say no recurrence since operation. |
| | | | | *Cases operated on after Nov.*, 1899. | |
| 31 | Mrs. A. 40 | Dec., 1899 | Mr. Carter ... ... | Very large, involving pectoral muscle and supra-clavicular glands. | Rapid recurrence in chest wall. |
| 32 | Mrs. Y. 69 | April, 1900 | Mr. Martin, of Temple Cloud | Growth in each breast. Both breasts removed at interval of five weeks. | Recurred on one side ten months later as nodules in subcutaneous tissue and pectoral muscle, and after their removal as extensive skin growth and died two years after first operation. |
| 33 | Mrs. W. 47 | July, 1900 | Mr. J. Dacre ... | Small growth. Scirrhus growth in glands. | Mr. Dacre reports no local recurrence, but died two years after operation from "growth in lungs." |
| 34 | K. L. 40 | July, 1900 | Children's Hospital | Small ... ... ... ... | Patient writes to say now free from recurrence, two years and three months after operation. |
| 35 | J. H. 64 | June, 1900 | Women's Ward of Children's Hospital | Small growth ... ... ... | Patient says now free from recurrence, two years and five months after operation. |
| 36 | Mrs. A. 48 | Sept. 1900 | Dr. Belfield ... | Extensive growth, and skin much involved. | Early recurrence. Died two years after operation. |
| 37 | C. P. 70 | Nov., 1900 | General Hospital.. | Moderate size ... ... ... | Patient writes to say now quite well, two years since operation. |
| 38 | Mrs. T. 58 | Dec., 1900 | Mr. A. W. Peake.. | "Adeno-fibroma" removed by another doctor eighteen months before. Scirrhus nodules near scar and growth in glands. | Patient now writes to say no recurrence. |
| 39 | Miss B. | Feb., 1901 | Dr. E. Aust Lawrence. | Two small scirrhus nodules in same breast. | Examined by me. No recurrence now, one year and ten months since operation. |

| NO. | NAME AND AGE. | DATE OF OPERATION. | SENT TO ME BY. (Where the patient was not specially sent to me, the name of Hospital in which operation done is given.) | CONDITION OF GROWTH. | RESULT. |
|---|---|---|---|---|---|
| 40 | Mrs. T. | Mar., 1901 | Mr. Martin, ... | Moderate size ... ... ... | Died a year later from "abscess in brain." |
| 41 | Mrs. V. 37 | April, 1901 | Dr. Nicholson ... | Small growth. Micro. section showed cancer. | Dr. Nicholson reports no recurrence now, one year and six months after operation. |
| 42 | L. W. | July, 1891 | General Hospital.. | Large growth ... ... ... | Recurrence a few months after operation in subcutaneous tissue and surface of pectorals |
| 43 | E. P. 39 | Oct., 1891 | Mr. A. W. Peake.. | Moderate size ... ... ... | Mr. Peake now reports no recurrence. |
| 44 | J. C. 56 | Feb., 1901 | Dr. Belfield .. | Large growth ... ... ... | Recurrence observed a year later attached to pectoral muscle. |
| 45 | Mrs. B. 38 | Oct., 1901 | Mr. Brew ... | Moderate size ... ... ... | Mr. Brew reports no recurrence now, one year after operation. |
| | | | | *Cases operated on within the last* | *year.* |
| 46 | Mrs. B. 53 | Jan., 1902 | Mr. A. W. Peake.. | Moderate size. Many small cancerous glands removed from above clavicle. | Died from secondary growth in liver, July, 1902, without local recurrence. |
| 47 | E. C. 58 | Feb., 1892 | Dr. Barker ... | Large growths and many glands... | Recurrence two months later. |
| 48 | Mrs. P. 60 | Feb., 1892 | Dr. J. Young ... | Moderate size ... ... ... | Recurred three months later as nodule attached to axillary vein: removed; then recurred in skin at back of axilla and widespread area of skin and supra-clavicular glands. Now small recurrence. |
| 49 | H. J. 41 | Mar., 1892 | General Hospital.. | Large growth ... ... ... | |
| 50 | M. H. 38 | April, 1902 | General Hospital.. | Large growth, involving muscle. Pectoral removed and also supra-clavicular glands. | |
| 51 | H. S. 50 | April, 1902 | General Hospital.. | Moderate size ... ... ... | |
| 52 | J. Y. 53 | April, 1902 | Dr. Hardman, .. | Moderate size ... ... ... | |
| 53 | H. S. 46 | Aug., 1902 | General Hospital. | Moderate size ... ... ... | |
| 54 | Mrs. B. 69 | Oct., 1902 | Mr. L. M. Griffiths | Moderate size. Degeneration cyst. | |

NOTE.—I have portions of growth from a large number of these cases still preserved in spirit, but have not yet had time to get sections prepared and examined.

this kind is, however, encouraging; for with a very large growth in which a degeneration cyst had formed, and over which the skin was of a red colour, there is no recurrence at the present time, 3 years and 3 months after the operation. As I have had no death from the operation, even in the most extensive cases, and as occasionally even a very extensive case does very well after operation, we should, I think, operate so that they may have a chance of prolongation of life if they understand that a cure is not probable. I have never refused to operate on account of the size of the growth or the extent of the affection of the axillary glands, and I do not hesitate to excise the axillary vein with the cancerous glands in cases in which it is actually involved in the growth. Very widespread diffusion of nodules in the skin I should regard as a contra-indication, and until the last few years I have so regarded the presence of supra-clavicular glands, but I should now remove them. Fixation to the chest wall would also deter me from operating; not so, however, fixation to the pectoral muscle, as I should in such cases remove the whole of the sternal portion of the muscle. Even with the arm fully raised so as to put the great pectoral muscle thoroughly on the stretch, I have found that in a few cases, though the breast may appear not to be fixed to the great pectoral, yet when the operation is done, it is found to have either invaded the muscle or be firmly adherent to it. I do not now, therefore, ever feel sure before I operate that the pectoral muscle is not involved. Sometimes under the anæsthetic the pectoral can be so stretched that fixation can be discovered when it was not before.

I do not think we can regard a history of the short duration of the growth before operation as of any value for prognosis, for in many cases the patients may have had the growth for a long time before they accidentally noticed it; but the fact that the patient had known of the existence of the growth for some considerable time before its removal, in the cured cases, would be of interest. I find on looking through my records that in two cured cases the growth had been noticed for a year, and in the others for only a few months or even less.

In some of my cases of very extensive growth there has been shock at the time of the operation, but it has been very exceptional; and in my oldest cases (No. 6 æt. 68, No. 29 æt. 73—a growth very extensive, No. 37 æt. 70, No. 54 æt. 69) it has not been present in any one. Indeed at the age of about 70 several of my patients have gone through this operation as a woman of 40 might be expected to do; and in one case, No. 32, a lady of 69 had both breasts removed with an interval of five weeks, and was none the worse for either operation. Many of these operations take more than two hours, if we include the final preparation of the skin under the anæsthetic (Halsted says his operation takes two to four hours), and yet shock is quite the exception. I would commend this fact to the attention of those who try to rush through an operation, and time it as if it were a record-breaking cycle performance. No one can more strongly object to waste of time in operating than I do, but I as strongly object to that rush and hurry which I do not believe is compatible with the best work. I much more fear loss of blood than I do an extra half-hour or even hour in operating. I know of few more extensive operations than the operation for cancer of the breast ought to be, (Mr. Butlin compares it to amputation through the upper third of the thigh as regards severity), and yet one may often spend two hours over it, and the patient is none the worse. I do not mean that all cases take two hours, but I never trouble if they require more.

I will now describe the method of operating I have employed in these 54 cases. I remove the skin over the breast, and always thoroughly around the tumour itself whether it involves the skin or not. Thus if the tumour is on the sternal side of the breast a very irregular shaped incision may be required. The pectoral fascia is always peeled off with the breast, and if not then completely removed, any portions left are carefully dissected away. I frequently take some surface fibres of the muscle rather than leave any fragments of the fascia. The whole fatty contents of the axilla (containing glands in many cases) are removed. I first define the axillary vein, and then clear out the axilla from above downwards and outwards. No division is made between the breast and the axillary contents, and thus no glands or lymphatics containing cancer cells are cut across.

In nearly all cases I save the long subscapular nerve, but remove the corresponding vessels with the fatty tissue. The fascia and fat between the two pectorals are also removed, and occasionally a nodule of growth is found here. Since 1897 I have made the dissection out of the fatty tissue around the breast more extensively in the midsternal and clavicular directions. There is, of course, nothing original in this operation, but I think it is important to state exactly what I do in considering the results of operation.

I have not adopted Halsted's method of removing the great pectoral muscle unless it is involved in the growth, or the growth is fixed to it; nor his plan of removing the fatty tissue from the posterior triangle in all cases, though I have begun to do so now if I find the highest infra-clavicular glands are infected. I have already stated that I do not regard the presence of such glands, discovered before operating, as a contra-indication to operation, and that I should remove them. Halsted has recorded two cases in which there was no recurrence for three years after their removal at a secondary operation, and therefore I think the outlook in such cases is by no means desperate.

I have carefully looked through my records to ascertain exactly where the recurrences took place, and I find that, unless the nodule in case 48 was a gland, and I do not think it was, there has been no recurrence in the axillary glands, for they have all been removed. In a few cases there has been recurrence in the supra-clavicular glands and once in the sternum. The latter could not be avoided by any method of operating, and should be regarded as a distant metastasis. In the great majority of cases the recurrence has been in the skin, subcutaneous tissue over the great pectoral or in the surface of the muscle itself. When we find recurrent nodules of growth in the subcutaneous tissue involving the surface of the muscle, it is impossible to say in which structure they started. So far then as these observations go, they point to the need of more complete removal of the skin and of either the surface of the muscle, to get rid more thoroughly of the pectoral fascia, or of the sternal portion of the muscle. We must remember when we get recurrences in the skin over the area where the breast has been removed, that skin may be axillary skin, or skin over the sternum or elsewhere, dragged into the area from which the breast has been removed at the time of operation, after being extensively separated from the underlying parts. The fact that in several cases the supra-clavicular glands were involved points to the advantage of a systematic removal of glands from above the clavicle at the first operation, as Halsted now does, even though they cannot be felt to be enlarged.

